# Home is where the home range is: Identifying territoriality and exhibit preferences in an ex-situ group of all-male Nile crocodiles (*Crocodylus niloticus*)

**DOI:** 10.1371/journal.pone.0297687

**Published:** 2024-01-25

**Authors:** Laura Stalter, Megan Terry, Alex Riley, Austin Leeds

**Affiliations:** Animals, Science and Environment, Disney’s Animal Kingdom^®^, Lake Buena Vista, FL, United States of America; University of Liverpool, UNITED KINGDOM

## Abstract

Here, the presence or absence of territoriality was evaluated in an all-male Nile crocodile (*Crocodylus niloticus*) group living in an ex-situ environment. Location data for each crocodile within the exhibit were collected three times per day over a two-year period, including two warm seasons and two cold seasons. A geographic information system (GIS) was used to create seasonal home ranges and core areas for each crocodile, to quantify the overlap of these home ranges and core areas to assess potential territoriality, and to calculate exhibit preferences of the group. Core area overlap was significantly lower than home range overlap, suggesting the crocodiles established territories within their exhibit. This pattern of behavior was similar across seasons, though it moderately intensified during the cold season. The crocodiles appeared to be more territorial in water, as overlap was most concentrated on the central beach, the only feature utilized more than expected based in its availability in the exhibit. These findings highlight the behavioral complexity of Nile crocodiles in human care, specifically the ability of Nile crocodiles to adapt to ex-situ environments similar to their wild counterparts by forming territories despite spatial constraints. Identifying the presence of territorial behavior is important for the care and welfare of ex-situ animals, as territorial animals have specific requirements that may result in increased agonism when unmet. It can also provide valuable context to aid in mitigation strategies, for example, when undesirable levels of agonism do occur. The findings here provide an example of how methodology from the wildlife ecology field can be adapted to ex-situ settings using a GIS and contributes to the current understanding of crocodilian behavior in human care.

## Introduction

Territoriality, the defense of an area against conspecifics, is a behavior observed across the animal kingdom [1]. Animals defend territories through indirect behaviors, such as visual, acoustic, and olfactory cues [2,3] and through direct encounters with conspecifics, such as agonistic interactions [4]. Access to resources drives territoriality, and the resources an animal chooses to defend within a territory vary by species [5–12]. For example, certain species may defend territories at specific times of the year surrounding seasonal resources, including during breeding seasons when access to mates, breeding sites, and nesting sites are competed for [13] or during times of food scarcity [14]. Territories can also be formed around more permanent ecological features, including water and shelter [13], or even more discrete taxa-specific resources, such as basking sites that serve as an essential resource for thermoregulation and ultraviolet B (UVB) absorption in reptiles [15,16]. Studying how territorial species prioritize the resources in their environment contributes to understanding their natural history and is essential for both in-situ [e.g. 17] and ex-situ [e.g. 18] animal management.

Territoriality can be identified on an individual level, in which an animal defends a specific area, and on a population level, in which multiple individuals of a species spatially partition out discrete regions within a single habitat [3]. Therefore, studying the spatial organization of animals, or how animals arrange themselves within a space, is key to revealing patterns of territoriality. Home ranges and core areas are two common measures used to describe animal space use at the individual level [19–22]. A home range encompasses the whole area that an individual has been known to spend time, while a core area represents a smaller range where an individual concentrates their time [1]. The overlap of home and/or core range measures can be used to quantify territoriality at the population level [23]. Since territorial animals exhibit spatial heterogeneity, they will have little to no core area overlap, while animals that are not territorial will distribute themselves more evenly throughout their space and display a similar amount of overlap between home ranges and core areas [24]. However, there is currently no agreed upon threshold of space use overlap that validates or rejects territoriality [14]. Furthermore, territoriality exists on a spectrum, and can depend on different factors, such as social structure or resource availability [2], which can make it difficult to identify territoriality by solely focusing on exclusive core areas. For example, in some species and populations, only the males are territorial [25] or a hierarchy may exist in which only certain dominant individuals maintain a territory [26]. In high density situations, including ex-situ environments with spatial constraints, it can be difficult to maintain exclusive territories as discretely as in-situ environments [27]. A more standardized method of identifying territorial patterns was proposed by Schlichting et al. [28], which resolves some of these limitations in identifying territoriality. In this approach, territorial animals are expected to show a gradual decrease in overlap when comparing home range to home range overlap (HR-HR), home range to core area overlap (HR-CA), and core area to core area overlap (CA-CA), while nonterritorial animals are expected to show approximately equal amounts of overlap at each of these space use overlap levels [1,2,28–31]. This method removes the arbitrary thresholds required to confirm exclusive core areas, leaving room for the realistic possibility that some level of core area overlap can occur in a territorial population.

Understanding the role of territoriality in ex-situ animals is important to meet optimal standards of care and welfare [32]. In ex-situ environments, an animal’s habitat is typically smaller than that of its wild counterpart so resources such as food, water, and key habitat features are more concentrated [33], and the distribution and availability of these essential resources can greatly impact how territorial animals will respond to their environment. If resources prioritized in an animal’s territory are not abundant and/or evenly distributed in an exhibit, it can be difficult for multiple individuals to maintain appropriate territories without increased agonism and can lead to welfare concerns. For example, Thomas et al. [34] investigated unusually high levels of agonism observed in a group of prairie dogs (*Cynomys ludovicianus*) and found that the colony split into two distinct groups that were competing for a concentrated food source. Providing a more even distribution of food proved to be a successful mitigation technique that significantly reduced aggressive behaviors. Additionally, Mechkour et al. [35] calculated home range and core area sizes for springbok (*Antidorcas marsupialis*) in an ex-situ environment and found that males with sufficient space and a consistent food supply were able to maintain territories similar to their wild counterparts. If a species is identified as territorial, planning exhibit modifications that allow individuals to maintain separated territories [18] and providing evenly distributed resources [34] is essential. Identifying how territorial animals adapt to zoological environments, with a focus on space and resource provision, is necessary for their management in human care but has not been well studied in a diversity of taxa.

Territoriality in Nile crocodiles (*Crocodylus niloticus*) is not well understood, however, available data suggest the intensity of territorial defense can vary by sex, season, and habitat type [36–39]. Basking sites and shallow water are important factors in Nile crocodile habitat choice, as crocodiles typically move in and out of water to thermoregulate throughout the day [38,40]. During the breeding season, home ranges are concentrated near basking and breeding sites, while foraging sites are prioritized outside of the breeding season [36]. Kofron [37] observed crocodiles congregating at basking sites without clear spatial partitioning, while territorial behavior was noted in water. Modha [41] similarly observed a group of Nile crocodiles basking together on land, but also found dominant males defending basking sites. Males may be more territorial than females based on spatial overlap [42], and a more detailed study of Nile crocodile spatial ecology found a lack of overlap between the home ranges of large adult males [36], suggesting a level of territoriality. Dominance hierarchies are common in territorial species [43] and previous studies also propose that Nile crocodiles form dominance hierarchies both in-situ and ex-situ [42,44]. If animals exhibit territoriality in human care, they require a sufficient space that allows them to express species-appropriate behaviors while maintaining a level of spatial avoidance with conspecifics [45,46]. For Nile crocodiles, this means being able to move in and out of the water to thermoregulate at appropriate basking sites without frequent risk of agonistic interactions from conspecifics defending territories. Territorial behavior likely occurs in ex-situ Nile crocodile populations, however, to our knowledge, no detailed evaluations of such phenomena have occurred. As Nile crocodile are managed in many settings, including zoos, aquariums, farms, and rescue centers, understanding territoriality in ex-situ environments is important to provide optimal care and welfare.

Disney’s Animal Kingdom^®^ cares for a large all male group of Nile crocodiles. Observations of this group to-date have identified seasonal patterns of agonistic behavior in this group [Disney’s Animal Kingdom^®^, unpublished data], for which territoriality has been suggested as a central driver. However, the presence of territoriality in this group has yet to be established. The purpose of this study was to provide a quantitative analysis of their space use to better understand if territorial behavior is occurring in this group, which can ultimately inform on Nile crocodile behavior and the care and welfare of the group. Specifically, we set out to:

1. Describe core area and home range sizes overall, by season and body size, using a GIS (geographic information system).
2. Quantify overlap between space use levels (HR-HR, HR-CA, CA-CA) with the utilization distribution overlap index (UDOI) and degree to identify the presence or absence of territorial behavior by season, space use level, and body size.
3. Quantify exhibit preferences for the group using an electivity index and compare variation by season and time of day.

To our knowledge, this is the first study to evaluate crocodilian space use in an ex-situ setting using a GIS, a powerful spatial analysis tool currently underutilized in ex-situ research [47]. We hope this study provides relevant insights for the behavior and management of ex-situ crocodilians and offers an example of how GIS can be used to advance the care and welfare of a variety of taxa living in human care.

## Methods

### Ethical note

This study was observational, non-invasive, and data were collected by the crocodiles’ animal care team as part of their day-to-day care of these animals. The methods of this project were approved by the scientific review committee at Disney’s Animal Kingdom^®^.

### Study subjects and housing

Study subjects included 21 Nile crocodiles living at Disney’s Animal Kingdom^®^ Theme Park, Lake Buena Vista, Florida. The group consisted of adult males between 32 and 38 years old at the start of the study, all of whom have lived together as a group at Disney’s Animal Kingdom^®^ since 1997. Individuals weighed between 219–405 kg. The number of crocodiles present varied from 21 individuals at the start of the study to 18 individuals at the end of the study. The crocodile exhibit was outdoors and contained islands, beaches, and open water maintained at an average 77.8°F (SE = 0.07). The exhibit was located along the path of a safari themed experience where guests viewed the exhibit from a truck (see Riley et al. [48] for additional exhibit details).

### Data collection

Between October 2020 and September 2022, the crocodiles’ animal care team collected data in support of this project as part of their daily record keeping. Data were collected by conducting a scan during which the location of each visible crocodile was marked on a map of the exhibit (S1 Fig). To coincide with the animal care team’s schedule and because previous research conducted on this group showed variation in behavior between morning, midday, and afternoon [48,49], scans were scheduled three times per day between 6:30am-9am, 10am-12:30pm, and 3pm-5:30pm. Due to the variability in day-to-day animal care, it was not always possible to conduct all three scans each day throughout the entire study. A total of 1,286 scans were conducted over the two-year period, with an average of 1.8 scans per day. The locations were then manually digitized onto a 600x600 grid overlaid with the same exhibit map in the ZooMonitor application [50]. An outline of the exhibit was created in ArcGIS Pro [51] using a combination of references, including satellite imagery, a blueprint of the exhibit, and manual measurements (see S1 File and S2 Fig for supporting materials). Locations were then imported into ArcGIS Pro [51] and georeferenced using an affine transformation to determine their accurate map coordinates (see S2 Table for location data). Seasonality in the crocodile’s behavior has been observed in this group [Disney’s Animal Kingdom^®^, unpublished data] and thus this observation period encompasses two complete seasonal cycles (cold season, October-March; warm season, April-September). Data were further grouped into four sampling periods, defined as a single season by year (e.g. Cold Season 1 = cold season, year 1).

### Home ranges and core areas

Kernel density estimates (KDE) [52] were derived in ArcGIS Pro [51] for each of the four sampling periods. The number of individuals, and therefore the number of KDE’s estimated, varied by sampling period. Two common bandwidth (i.e. smoothing parameter) selectors were tested, least square cross validation and href, which resulted in over- and undersmoothing, respectively. Therefore, the bandwidth was determined using the optimal bandwidth function,

$$h_{opt}{=[\frac{2}{3n}]}^{\left(\frac{1}{4}\right)}\sigma ,$$

where *n* is the sample size (number of observations) and *σ* is the standard distance of locations [53,54]. To avoid including inaccessible areas when calculating KDE, a polygon of the exhibit’s boundary was used as a barrier. The 95% fixed-kernel method was used to define the home range (HR), which contains 95% of all recorded locations, and the 50% fixed kernel method was used to define the core area (CA), which represents the area where the animal’s use is most concentrated [55–58]. The area (m^2^) of each home range and core area generated was calculated in ArcGIS Pro [51].

### Overlap

Measuring spatial overlap is a useful method for evaluating spatial tolerance or avoidance, and ultimately territoriality [28]. A utilization distribution overlap index (UDOI) is a particularly useful index to quantify spatial overlap because it accounts for each individual’s probability density at a given point [23]. UDOI increases when both individuals’ locations are highly concentrated in the same space. UDOI ranges from 0 (no overlap) to 1 (full overlap) but can be > 1 if high overlap exists and the utilization distributions are non-uniform. The UDOI was estimated to measure the amount of spatial overlap between each dyad for each sampling period at all three levels of space use (HR-HR, HR-CA, and CA-CA) with the formula:

$$UDOI=A_{i,j}\int_{x}\int_{y}{UD}_{i}(x,y)\times {UD}_{j}(x,y)$$

[23,28,59]. *A*_*i*,*j*_ is the area of intersection between both animals’ home ranges. *UD*_*i*_(*x*,*y*) is the value of animal i’s utilization distribution at a given cell. *UD*_*i*_(*x*,*y*)×*UD*_*j*_(*x*,*y*) is the integrand, or the cell-by-cell product, of both utilization distributions. $\int_{x}\int_{y}{UD}_{i}(x,y)\times {UD}_{j}(x,y)$ refers to the normalized integration of these values. All components were calculated in ArcGIS Pro [51]. We also calculated each individual’s degree at each sampling period and space use level by summing the number of conspecifics individual crocodiles overlapped with [28,60,61]. When calculating degree, dyads with UDOI ≥ .01 were considered to be overlapping. For HR-HR and CA-CA overlap, degree was one-directional. For HR-CA overlap, degree was calculated as the number of home ranges that overlapped with an individual’s core area.

Network diagrams were generated in the package “igraph” [62] in R V.4.2.2 [63] to visualize the UDOI and degree measurements between individuals. For simplification, network diagrams only include individuals who were present in each of the four sampling periods (n = 18) with edge weights representing an average (UDOI) or binary (degree) value at the season level. Node distribution was fit using a Fruchterman-Reingold force-directed layout. Placement of nodes within each diagram was based on a multidimensional scaling arrangement such that the distance between nodes was inversely proportional to their association index (UDOI or degree), or that strongly associated individuals or individuals with more associations were physically closer within the diagram. Edge weights were proportional to mean UDOI values and binomial degree values defined by the occurrence of overlap during a season.

### Preference

Electivity indices quantify space utilization by comparing the actual use of a zone to its expected use based on that zone’s availability in the exhibit. Zones that are used in greater proportion than its availability, or overutilized, are typically considered preferred and it may be beneficial to increase the abundance of these desirable features in the exhibit. Underutilized spaces could indicate less desirable features or those features may be more abundant than necessary. A neutral value typically indicates that those features are adequately provided for the species or individual.

**Fig 1:**
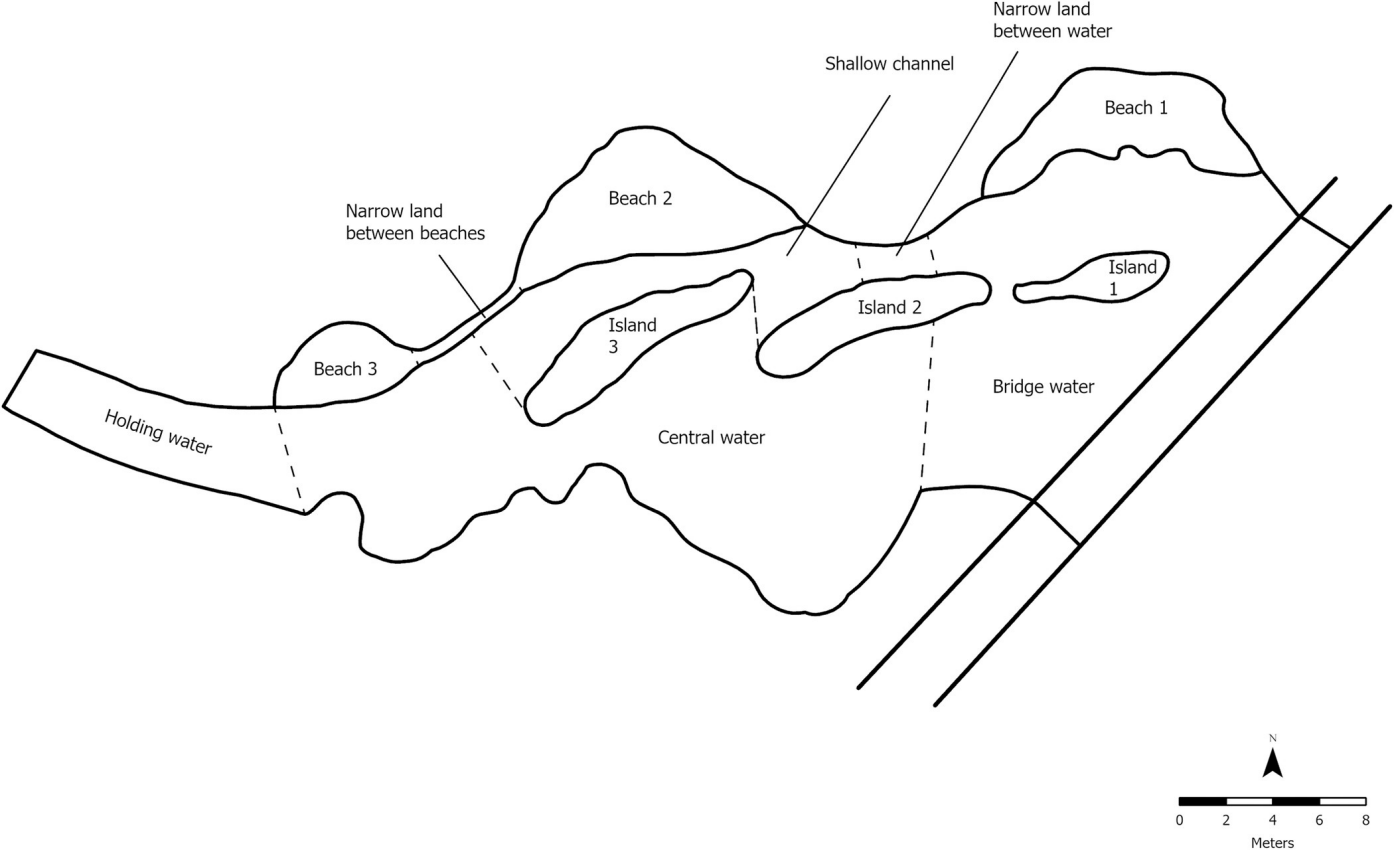
Nile crocodile exhibit labelled by exhibit feature. For a description of exhibit features, see Table 1.

The crocodile exhibit was divided into individual zones, or “features” (see Table 1 and Fig 1). Each island and beach were categorized as a distinct feature and open water was divided into sections to account for the distance from the bridge and from the off-show holding area. The area of each feature was calculated in ArcGIS Pro [51] to get the proportion of each feature in relation to the whole exhibit, which was used to calculate “expected values”. Each crocodile location was assigned an exhibit feature to obtain the “actual values”. We used the electivity index of Vanderploeg and Scavia [64] to analyze preference:

$$E*=\frac{W_{i}-(\frac{1}{n})}{W_{i}+(\frac{1}{n})}$$

where r_i_ is the observed use (proportion of locations) of feature i, p_i_ is the expected use (proportion of locations), *n* is the number of exhibit features, and

$$W_{i}=\frac{\frac{r_{i}}{p_{i}}}{\sum \frac{r_{i}}{p_{i}}}$$

[65,66]. E* was determined for each crocodile during each sampling period and for each crocodile at each time of day.

**Table 1:** Nile crocodile exhibit features.

Area	Code	Description	Size (m^2^)	% Exhibit
Beach 1	B1	Grassy beach adjacent to bridge	34.10	5.49
Beach 2	B2	Beach adjacent to shallow channel covered with dirt substrate in the center of the exhibit	45.60	7.35
Beach 3	B3	Beach closest to holding with dirt substrate	15.30	2.47
Bridge water	BW	Open water adjacent to bridge	183.02	29.49
Central water	CW	Open water in the center of the exhibit	200.37	32.28
Holding water	HW	Narrow strip of water that leads to off-exhibit holding area	41.58	6.70
Island 1	I1	Grassy island adjacent to bridge	8.59	1.38
Island 2	I2	Grassy island connected to narrow land	20.02	3.23
Island 3	I3	Grassy island adjacent to shallow channel	21.74	3.50
Narrow land between beaches	NLB	Small strip of land that connects beach 2 and beach 3	5.29	0.85
Narrow land between water	NLW	Small strip of land between water by the bridge and shallow channel	3.54	0.57
Shallow channel	SC	Narrow strip of shallow water surrounded by other land features	41.50	6.69

### Analysis

Generalized linear mixed models were run using the function glmmTMB [67,68] in R Studio [63,69] to analyze patterns in home range size and territoriality. Full models with all a priori fixed factors included were run rather than conducting step-wise model fitting as the latter raises concerns over data dredging, multiple testing, and interpretive value of final models [70–72]. Collinearity within each model was assessed using a variance inflation factor (vif) test using the vif function. Post hoc comparisons were conducted using t tests with a Tukey adjustment for multiple comparisons.

To model factors influencing home range size, two models were run with a dependent variable of home range size and core area size, respectively, both fit with a Gaussian distribution. Season and individual weight were included as fixed factors. Season was included to evaluate if home range patterns varied by time of year and individual weight was included to evaluate if individual demographic variables influence space use. Crocodile identity and year of study were included as random factors to account for repeated sampling over the two-year study period.

To test for evidence of territoriality we used two dependent variables, degree and UDOI. For the UDOI model, our first fixed factor was space use level, or the overlap of HR to HR, HR to CA and CA to CA between all dyads [e.g. 28]. We additionally included season and weight as a fixed factors. We did not include population size as a fixed factor due to its small range throughout the study. Initially, the interaction of season and space use level was included as a fixed factor, however, we were met with issues of model convergence and multicollinearity, which was resolved by eliminating the interaction. Crocodile dyad and year of study were included as random factors. The UDOI model was fit with a zero inflated Gaussian distribution, as the data were positively skewed towards zero. Our first two degree models included space use level and weight as fixed factors. To control for differences in season, we ran a separate model for each season. We initially included season and space use level as fixed factors, however, these were eliminated to resolve issues of model convergence and multicollinearity. Because of these issues, and to see if degree varied by season, we ran a separate model with season as the only fixed factor. All degree models included crocodile identity and year of study as random factors and were fit with a Poisson distribution.

To test for individual feature selectivity, we calculated the percentage use per feature for each individual during the entire study period and compared the group’s mean observed percentage use to the expected percentage use using Mann-Whitney U-tests. To test for seasonal patterns of exhibit preferences, an electivity index was calculated per feature for each individual, each season. Wilcoxon signed rank tests with a continuity correction were then used to compare the mean electivity indices of each feature during the warm season versus the cold season. To test for differences in exhibit preferences at different times of day, an electivity index was calculated per feature for each individual at each time of day over the entire study period. Then the mean electivity indices of each feature during the morning, midday, and afternoon were compared using a Friedman test. Post hoc comparisons were conducted using a Wilcoxon test with continuity correction and p-values were adjusted using the Bonferroni method to account for multiple tests. Statistical analyses were not conducted for seasonal and time of day tests for island 1, as no crocodiles were recorded at that feature during the study. Significance for all tests conducted for this study was set to 0.05 and all model output values are presented as estimated marginal means (EMM) ± standard error (SE). Full statistical outputs are presented in S1 Table.

## Results

### Home range size

The average home range size across all individuals and all seasons was 91.58 m^2^ and the average core area size was 16.11 m^2^. Season significantly predicted home range size (*X*^*2*^ = 7.748, *df* = 1, *P* = 0.005). Home ranges were larger during the warm season (μ = 97.60, SE = 9.88) compared to the cold season (μ = 87.10, SE = 9.83) (Table 2). Core area size was not predicted by season (*X*^*2*^ = 0.125, *df* = 1, *P* = 0.724). Weight did not significantly predict home range size (*X*^*2*^ = 2.107, *df* = 1, *P* = 0.147) or core area size (*X*^*2*^ = 1.678, *df* = 1, *P* = 0.195).

**Table 2:** Home range and core area sizes (m^2^) of Nile crocodiles by season.

	Home Range					Core Area				
Season	n	EMM	S.E.	Min	Max	n	EMM	S.E.	Min	Max
Cold	39	87.10	9.83	31.18	209.95	39	16.20	1.69	5.41	45.55
Warm	37	97.60	9.88	23.70	244.14	37	15.90	1.70	3.27	45.10

### Overlap

**Fig 2:**
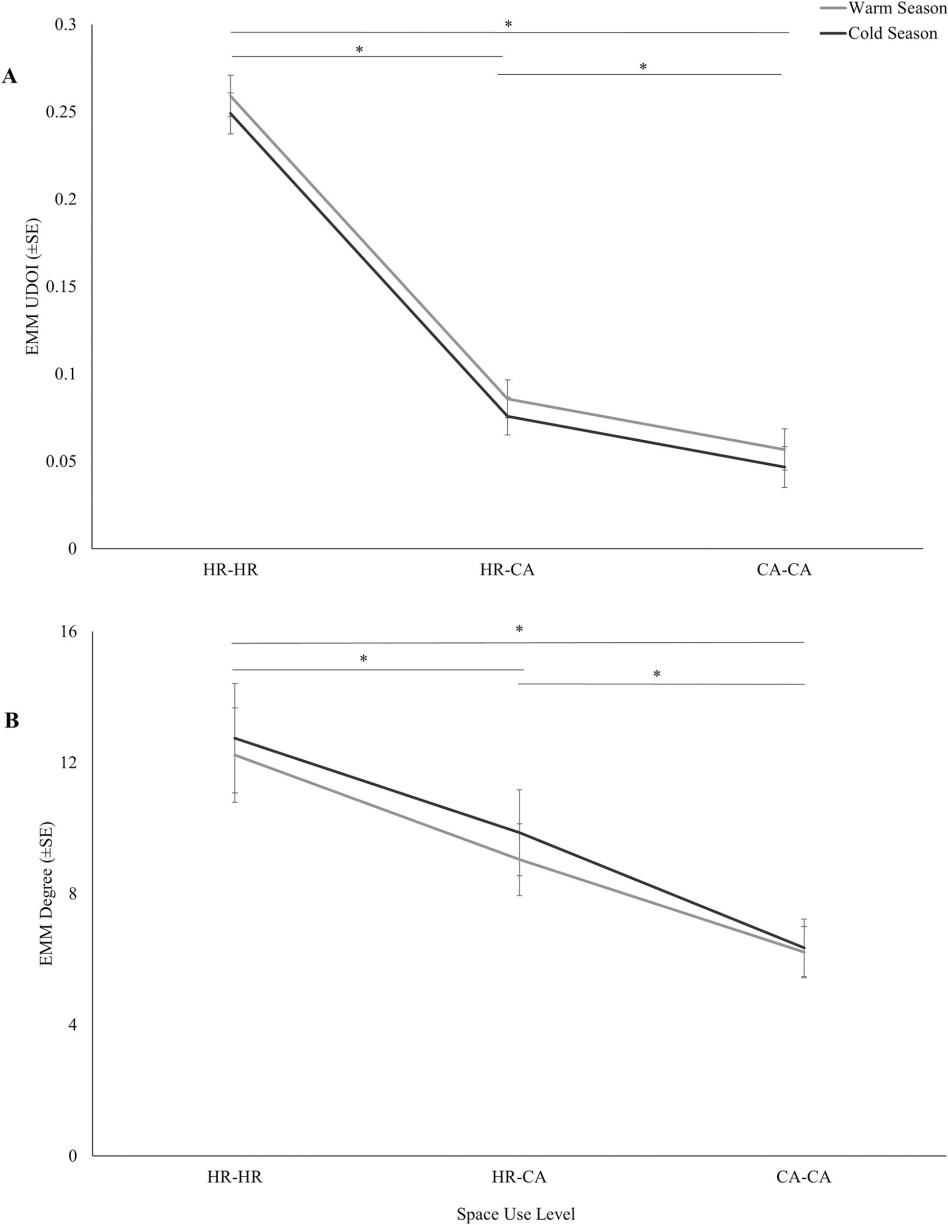
Utilization distribution overlap index (UDOI) (A) and degree (B) estimates for home range to home range (HR-HR), home range to core area (HR-CA), and core area to core area (CA-CA) overlap during the cold and warm seasons. The asterisk denotes statistical significance (P≤0.05).

**Fig 3:**
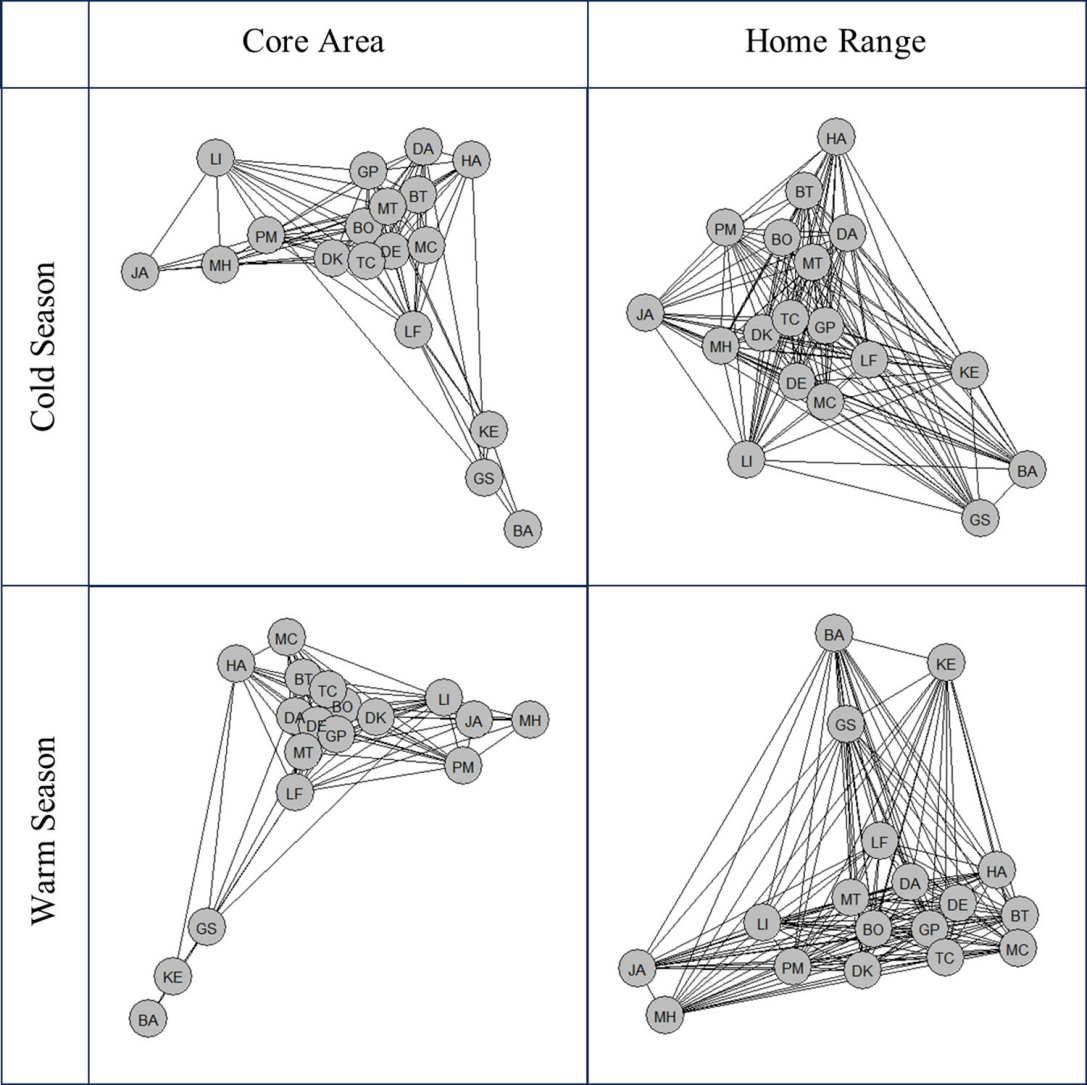
Network diagram of Nile crocodiles based on utilization distribution overlap index (UDOI). Circular nodes represent individual crocodiles.

The crocodiles’ space use overlap (UDOI) patterns indicated territoriality, as there was a sharp decrease between HR-HR overlap (Warm: μ = 0.259, SE = 0.012; Cold: μ = 0.249, SE = 0.012) and HR-CA overlap (Warm: μ = 0.086, SE = 0.011; Cold: μ = 0.076, SE = 0.011), and overlap continued to decrease between the levels HR-CA and CA-CA (Warm: μ = 0.057, SE = 0.012; Cold: μ = 0.047, SE = 0.012) (Fig 2A). UDOI was significantly different between space use levels (*X*^*2*^ = 1,006.401, *df* = 2, *P*<0.001), and post hoc comparisons of space use levels showed a significant difference in UDOI between each level: HR-HR and HR-CA (*t* = -25.885, *df* = 2,739, *P*<0.001), HR-CA and CA-CA (*t* = -4.341, *df* = 2,739, *P*<0.001), and HR-HR and CA-CA (*t* = -29.113, *df* = 2,739, *P*<0.001). Network diagrams showed varied levels of overlap between dyads, with some individuals consistently showing low overlap with the rest of the group, particularly in core area overlap (Fig 3). Space use overlap was significantly different between seasons (*X*^*2*^ = 3.932, *df* = 1, *P* = 0.047). UDOI was greater during the warm season (μ = 0.134, SE = 0.012) than the cold season (μ = 0.124, SE = 0.012). Weight did not significantly predict UDOI (*X*^*2*^ = 3.223, *df* = 1, *P* = 0.073).

**Fig 4:**
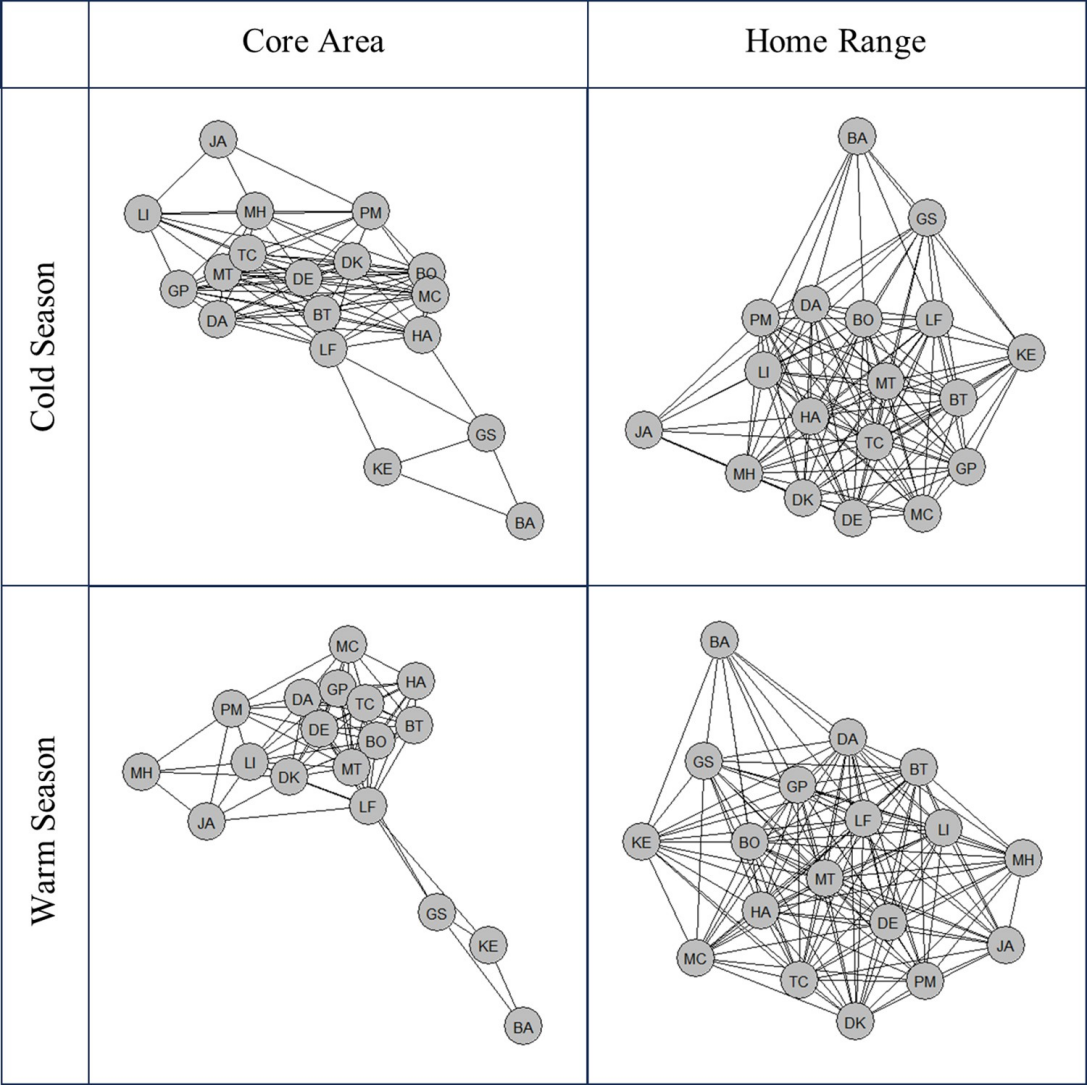
Network diagram of Nile crocodiles based on degree. Circular nodes represent individual crocodiles.

The crocodiles’ degree patterns also suggested territoriality within this group, as there was a decrease between levels HR-HR (Warm: μ = 12.230, SE = 1.438; Cold: μ = 12.750, SE = 1.669) and HR-CA (Warm: μ = 9.040, SE = 1.092; Cold: μ = 9.860, SE = 1.310) and levels HR-CA and CA-CA (Warm: μ = 6.220, SE = 0.783; Cold: μ = 6.350, SE = 0.875) (Fig 2B). Degree was significantly different among space use levels during both the cold (*X*^*2*^ = 88.342, *df* = 2, *P* <0.001) and warm (*X*^*2*^ = 74.705, *df* = 2, *P* < .001) seasons. During both seasons, post hoc comparisons between space use levels revealed a significant difference in degree between HR-HR overlap and HR-CA overlap, (Cold: *t* = -3.975, *df* = 111, *P*<0.001; Warm: *t* = -4.293, *df* = 105, *P*<0.001), HR-CA overlap and CA-CA ovelap (Cold: *t* = -5.648, *df* = 111, *P*<0.001; Warm: *t* = -4.487, *df* = 105, *P*<0.001), and HR-HR overlap and CA-CA overlap (Cold: *t* = -9.389, *df* = 111, *P*<0.001; Warm: *t* = -8.568, *df* = 105, *P*<0.001). Network diagrams showed variation in degree across individuals, especially in core area overlap (Fig 4). Season significantly predicted degree (*X*^*2*^ = 5.593, *df* = 1, *P* = 0.018). Degree was greater during the cold season (μ = 10.07, SE = 0.817) compared to the warm season (μ = 9.11, SE = 0.752). Weight significantly predicted degree during the cold season (*X*^*2*^ = 3.881, *df* = 1, *P* = .049), but not the warm season (*X*^*2*^ = 3.418, *df* = 1, *P* = 0.065). During the cold season, there was a 37% decrease in degree for every 10 kg increase in weight.

### Preference

**Fig 5:**
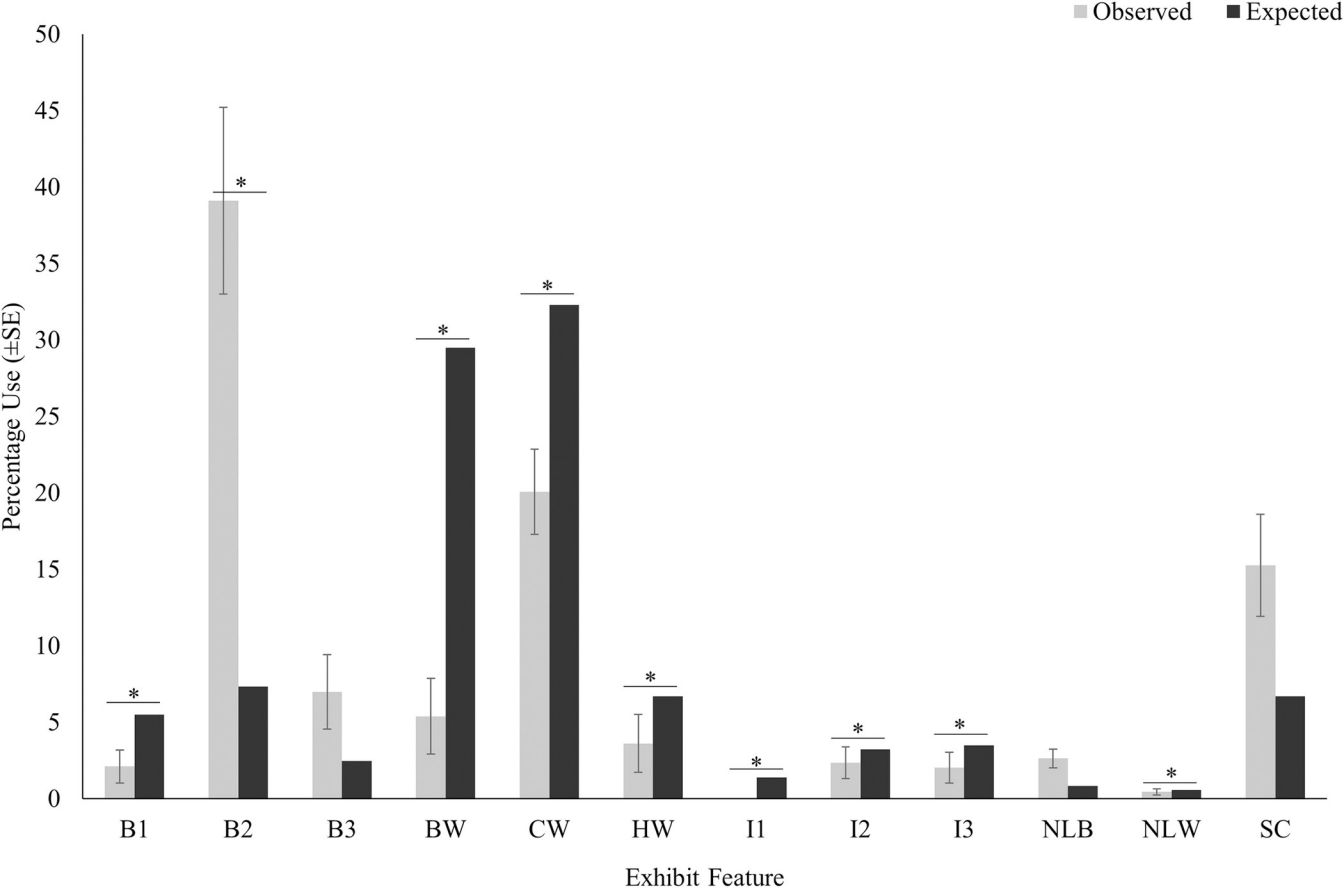
Observed and expected percentage use (Ave.±SE) for exhibit features. The asterisk denotes statistical significance (P≤0.05). See Table 1 for a detailed description of exhibit features.

Of all features, the crocodiles only showed a significant preference for beach 2 (*W* = 357, *P*<0.001) (Fig 5). The crocodiles significantly underutilized beach 1 (*W* = 84, *P*<0.001), the three largest water features (HW: *W* = 56, *P*<0.001; CW: *W* = 63, *P*<0.001; BW: *W* = 42, *P*<0.001), all three islands (I1: *W* = 0, *P*<0.001; I2: *W* = 84, *P*<0.001; I3: *W* = 63, *P*<0.001), and the narrow land between water (*W* = 84, *P*<0.001). Features used at rates proportional to their availability included beach 3 (*W* = 168, *P* = 0.178), the narrow land between beaches (*W* = 294, *P* = 0.052), and the shallow channel (*W* = 273, *P* = 0.173), as there were no significant differences between the observed and expected percentage use for these features.

**Fig 6:**
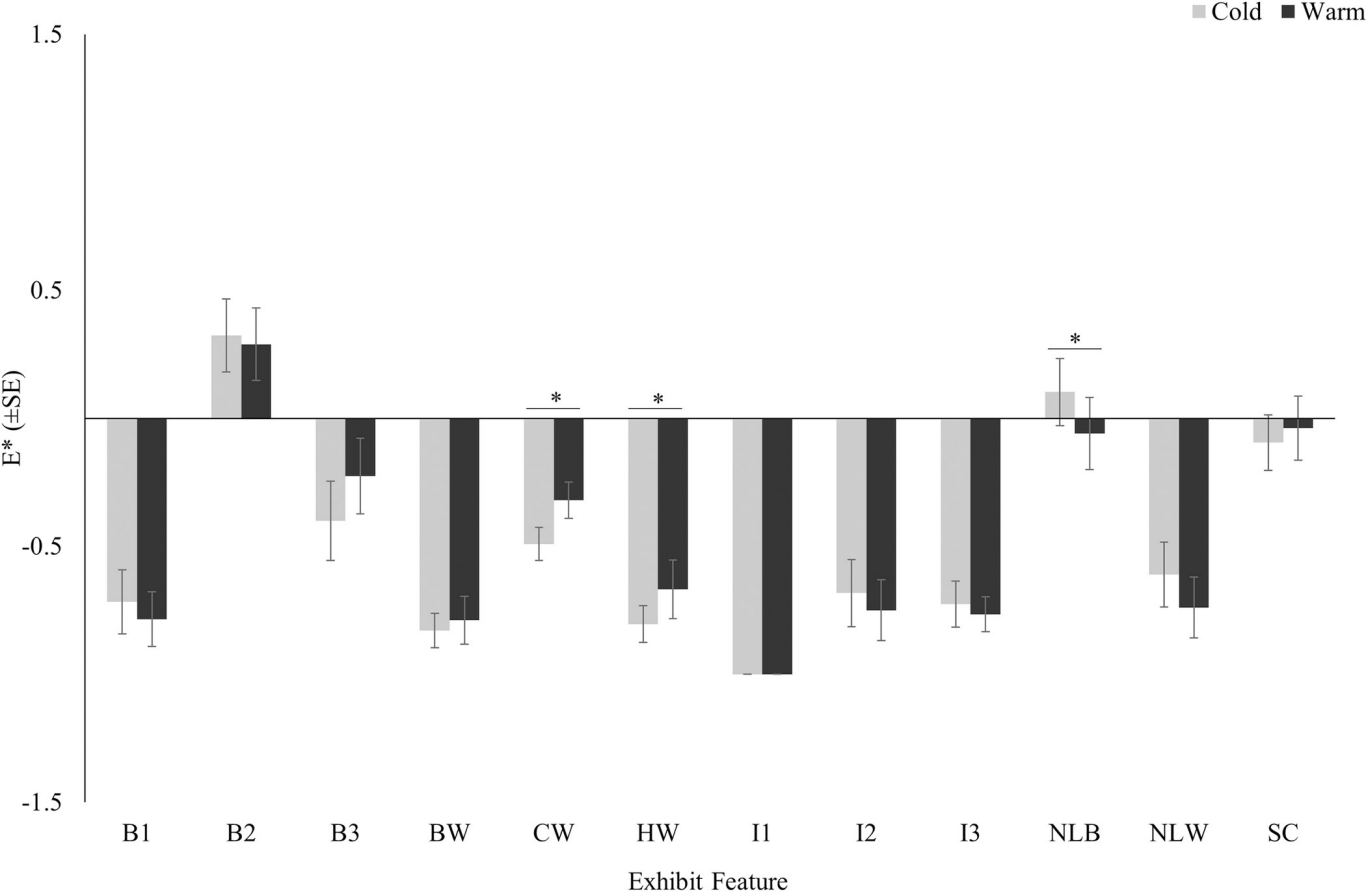
Electivity indices (Ave.±SE) for exhibit features by season. The asterisk denotes statistical significance (P≤0.05). See Table 1 for a detailed description of exhibit features.

There was a significant seasonal effect on the electivity indices for the central water (*V* = 7, *P*<0.001), the holding water (*V* = 10, *P* = 0.014), and the narrow land between beaches (*V* = 107, *P* = 0.047) (Fig 6). While still generally underutilized during both seasons, the crocodiles utilized the central water and holding water features less during the cold season compared to the warm season. The crocodiles utilized the narrow land between beaches more during the cold season compared to the warm season.

**Fig 7:**
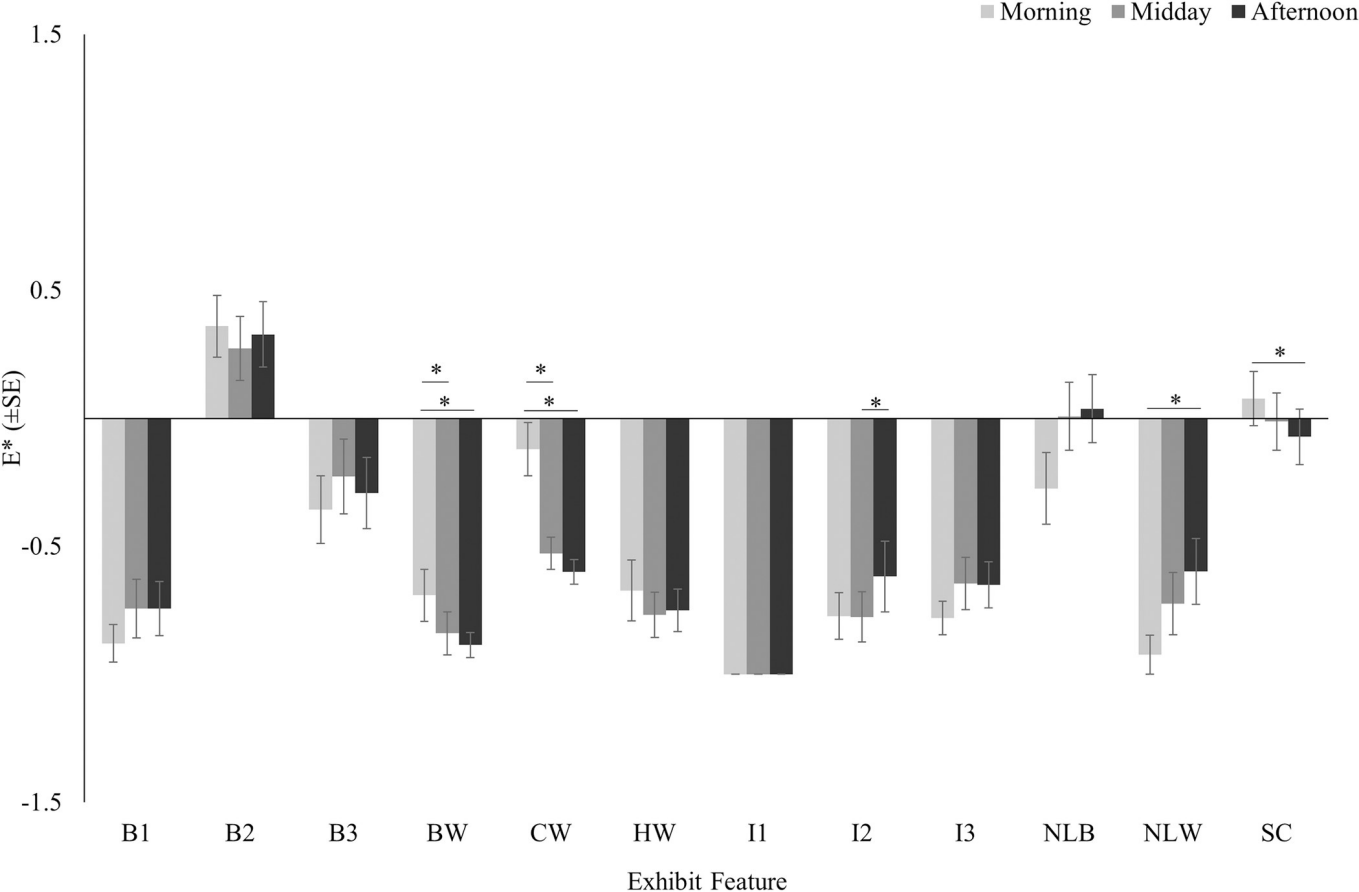
Electivity indices (Ave.±SE) for exhibit features at three times of day. The asterisk denotes statistical significance (P≤0.05). See Table 1 for a detailed description of exhibit features.

There was a significant effect of time of day on preference for the bridge water (*X*^*2*^ = 27.634, *df* = 2, *P*<0.001), the central water (*X*^*2*^ = 23.524, *df* = 2, *P*<0.001), island 2 (*X*^*2*^ = 9.941, *df* = 2, *P* = 0.007), the narrow land between water (*X*^*2*^ = 8.061, *df* = 2, *P* = 0.018), and the shallow channel (*X*^*2*^ = 10.381, *df* = 2, *P* = 0.006) (Fig 7). Post hoc comparisons revealed that the crocodiles utilized both the central water and the water by the bridge significantly more in the morning compared to midday (CW: *P*<0.001; BW: *P*<0.001) and in the morning compared to the afternoon (CW: *P*<0.001; BW: *P*<0.001). Similarly, the crocodiles utilized the shallow channel significantly more in the morning compared to the afternoon (*P* = 0.038). The narrow land between water was utilized significantly more during the afternoon compared to the morning (*P* = 0.043), and island 2 was utilized significantly more in the afternoon compared to midday (*P* = 0.043). The Friedman test also detected a significant effect of time of day for the narrow land between beaches (*X*^*2*^ = 7.177, *df* = 2, *P* = 0.028), but the post hoc test showed the difference in electivity indices approached but did not reach significance between morning and midday (*P =* 0.078) and between morning and afternoon (*P* = 0.061).

## Discussion

A GIS was used to create seasonal home ranges and core areas, to calculate overlap of these ranges, and to estimate exhibit preferences for a large, all male group of Nile crocodiles. Both metrics of overlap, UDOI and degree, significantly decreased between HR-HR overlap, HR-CA overlap, and CA-CA overlap. Space use overlap was lower during the cold season compared to the warm season. These results indicate the crocodiles established territories, and the intensity of this behavior was influenced by season.

In-situ studies have shown Nile crocodile spatial distribution varies by season [39,44]. Here, home range size was significantly larger in the warm season compared to the cold season, suggesting this ex-situ group similarly changes their space use patterns seasonally. The home range sizes of adult male Nile crocodiles estimated in-situ are magnitudes larger than the space available to the Nile crocodiles studied here [39,73], so it is notable that this behavioral pattern is adaptable in an ex-situ environment. The size of core areas, however, did not differ by season. As core areas are more restricted in space than home ranges, the crocodiles may not choose, or be able, to constrict core areas further. It is also possible that the crocodiles utilize a relatively consistent core area year-round but choose to maintain closer proximity to those core areas as opposed to moving more freely around the exhibit during the cold season. Body size did not predict home range or core area size. Given that all crocodiles in the study group are adult males of similar age, there may not be enough variation in weight to detect an influence of body size on the size of home ranges or core areas.

The patterns of space use overlap observed here are consistent with the patterns expected for territorial animals [28]. When comparing overlap between the three space use levels, home range overlap was highest and core area overlap was lowest, suggesting spatial avoidance among the crocodiles within their core areas. This finding has important implications for how Nile crocodiles are managed in human care. Territorial animals must be carefully managed, as there is a risk of increased agonism that arises with the drive to maintain territories. Decisions around group structure, such as introductions of new individuals, especially females, and changes in group size, may disrupt any stability of territories and lead to increased agonism as new territories are established. For example, in an effort to mitigate agonism in this group, individuals with low levels of core area overlap, which indicated high levels of territoriality, were separated from the group following this study. Territoriality should be accounted for when shifting (moving animals between on and off exhibit spaces) or changing feeding locations as well, as agonism may increase when animals move through the core areas of conspecifics or may be required to reestablish areas upon return. Another important factor to consider is the availability of key resources, such as food and basking sites, which should be widely distributed and allow sufficient space for individuals to spatially partition while meeting their daily needs. The abundance of resources necessary is dependent on group size, and the larger the group, the more widely dispersed resources should be. Another agonism mitigation strategy implemented in this group following this study included an exhibit renovation that increased the availability of shallow water and preferred beach space. Though it may be difficult to predict how a group of territorial animals will react to changes in their environment, careful attention should be given to the management of Nile crocodiles and the group should be closely monitored following any changes. When agonism does occur, these factors may be useful to consider when planning mitigation strategies.

At the individual level, some dyads exhibited no space use overlap between core areas, while other dyads showed variable levels of overlap. While it would likely be unrealistic to expect every individual to maintain completely exclusive core areas here or in nature, a few dyads showed much higher core area overlap than the group average. A more detailed look at the locations of core areas and home ranges compared to the locations of overlap may explain some of the higher core area overlap seen here. Core areas and home ranges were distributed across land and water features. However, almost all core area overlap and about half of home range overlap were located on land, specifically beach 2. Together, this suggests the crocodiles are more tolerant of conspecifics on land, and it is possible that the crocodiles maintain more exclusive territories in the water. Nile crocodiles in nature are known to bask together on land, seemingly subsiding any territorial behavior [37,74], and the crocodiles observed here show a similar pattern of behavior. Additionally, Kofron [37] observed agonism and dominance behaviors of wild Nile crocodiles primarily in the water, but not on shore, and a previous study of this group similarly found that the majority of agonistic behavior occurred in the water near beach 2 [49]. If the crocodiles are in fact more tolerant on land, as past research and findings here suggest, increasing land may reduce territoriality and associated behavioral consequences of territoriality, such as agonism and conspecific wounding, in ex-situ environments.

The patterns of space use overlap used to define territoriality were observed year-round in both the cold and warm seasons, but the extent of overlap differed by season for both metrics. UDOI overlap was lower during the cold season compared to the warm season, which would suggest increased territoriality during the colder months, further supported by the more concentrated home ranges during the cold season previously mentioned. Interestingly, this aligns with the increase in agonism observed in this group during the cold season [Disney’s Animal Kingdom^®^, unpublished data], of which territoriality may be a driving factor. In contrast, degree was higher in the cold season. Therefore, in the colder months the crocodiles overlapped less overall, but where they did overlap, they did so with more individuals. This is likely due to a change in exhibit use by season. The seasonal preference analyses showed that crocodiles used land more and water less during the cold season. Because the crocodiles are using the land more in the cold season and they seem to be more tolerant towards conspecifics on land, it then stands to reason that there would be a slight increase in the number of individuals with overlapping home ranges and core areas during the cold season. Additionally, larger individuals overlapped with fewer conspecifics during the cold season, so larger crocodiles may utilize space further from crowded areas and/or they may be actively defending core areas against smaller conspecifics during these months. Overall, seasonality does appear to influence this group’s territorial behavior. While the crocodiles exhibit territorial behavior year-round, it may intensify during the colder months, similar to broader seasonal changes in social behavior [48,49], which could help to contextualize the seasonal agonism observed in this group and should be broadly considered when making management decisions for any crocodilian, as they may respond to change differently depending on the season.

As a group, the crocodiles utilized beach 2, the largest, central beach, more than expected based on its availability in the exhibit, and the group utilized the adjacent shallow channel as expected based on availability. Calverely & Downs [38] suggest that the availability of suitable basking sites plays a significant role in Nile crocodile habitat choice in the wild. More specifically, Behangana et al. [40] found in-situ Nile crocodiles showed a preference for grassy river banks with easy access between the land and water with shallow water nearby where the crocodile can remain firmly planted on land while partially covered in water, features similar to the shallow channel and beach 2. Leigh and Brereton [73] similarly found that dwarf caimans in two zoological facilities preferred the shallow edges of water features where they can bask. During this study, crocodiles were never observed on island 1, perhaps due to the relatively steep incline required to climb on to the island or its proximity to the bridge. Due to its proximity to the shallow channel, it is notable that the group utilized island 3 less than expected as well. A preference at the group level may not have been detected due to the smaller size of island 3 compared to beach 2, which can hold more individuals at once. Aside from group preferences, individual differences were observed, including a few individuals who preferred the basking sites beaches 1 and 3, and islands 2 and 3. Because dominant individuals may monopolize certain habitat features in ex-situ settings [66], it is possible that these are more dominant individuals who defend these basking areas, but more research is needed. Increasing the availability of features used in greater proportion than expected is thought to have a positive effect on animal welfare in ex-situ environments [66], thus providing large stretches of beach space adjacent to gradual gradients of water within Nile crocodile exhibits may be an optimal design choice based on their natural history and behavioral preferences.

We found a significant difference in the preference for certain features by time of day. Here, the crocodiles used water features more in the morning and land features more midday and afternoon. Previous studies of this group [47,73] also found the crocodiles were in water more in the morning, and this is likely due to the need to bask during the day for thermoregulation and UVB absorption. Additionally, Leigh & Brereton [75] found a difference in behavior depending on time of day in zoo-housed dwarf caimans, with an increase in water-related behavior at night. Though this finding is not surprising, it highlights how strongly the crocodiles rely on specific exhibit features to meet their daily thermoregulatory needs. Since this group has been identified as territorial, this further supports the importance of ensuring sufficient availability and distribution of basking sites and water features, allowing crocodilians to fulfill their thermoregulatory needs.

### Conclusion

Through a combination of spatial and behavioral techniques, we offer a new context under which Nile crocodile social behavior can be understood. Specifically, we found that despite the spatial constraints of this ex-situ environment, the crocodiles exhibited territorial behavior that varied by season and by exhibit features. These findings provide additional insights into crocodilian behavioral complexity, a concept that is often overlooked in regards to the care and welfare of reptiles in ex-situ environments [76]. Therefore, to achieve optimal care and welfare, territoriality should be considered in the daily husbandry of Nile crocodiles, as territorial animals have specific space and resource requirements and territoriality may influence their behavioral response to changes in the environment. These findings also demonstrate that the use of spatial analyses often applied to wild populations can reveal similar adaptations ex-situ, which can help to better understand the needs of animals in living in human care, especially for animals with more cryptic behavior like crocodilians. We hope these findings contribute to the relatively small body of literature on ex-situ crocodilians and encourage the use of a GIS in ex-situ facilities to better understand and optimize the welfare of animals in human care.

## Supporting information

S1 Fig Crocodile exhibit map. This jpg file contains the hand-drawn map used in data collection and processing.
(JPG)

S2 Fig Crocodile exhibit outline map. This jpg file contains an outline of the crocodile exhibit over satellite imagery. This map was created using ArcGIS^®^ software by Esri. ArcGIS^®^ and ArcMap™ are the intellectual property of Esri and are used herein under license. Copyright © Esri. All rights reserved. For more information about Esri^®^ software, please visit www.esri.com.
(JPG)

S1 Table Generalized linear mixed model outputs for predictors of home range size (A), core area size (B), utilization distribution overlap index (UDOI) (C), degree during all seasons (D), degree during the cold season (E), and degree during the warm season (F). CA-CA refers to core area to core area overlap, HR-CA refers to home range to core area overlap, and HR-HR refers to home range to home range overlap. Bolded variables denotes statistical significance (P ≤ 0.05) for predictor variables. Parameter estimates with ‶-″ are compared to remaining variable conditions within each predictor variable.
(DOCX)

S2 Table Crocodile location data deposit. This csv file contains the original location data analyzed in the main text.
(CSV)

S1 File Crocodile exhibit outline. This zipped shapefile outlines the spatial boundary of the crocodile exhibit at Disney’s Animal Kingdom^®^.
(ZIP)
