# Supplementary figures and images for: Home is where the home range is: Identifying territoriality and exhibit preferences in an ex-situ group of all-male Nile crocodiles (Crocodylus niloticus)
Source: PLoS One. 2024 Jan 25;19(1):e0297687. doi: 10.1371/journal.pone.0297687 (PMC10810454; doi:10.1371/journal.pone.0297687)

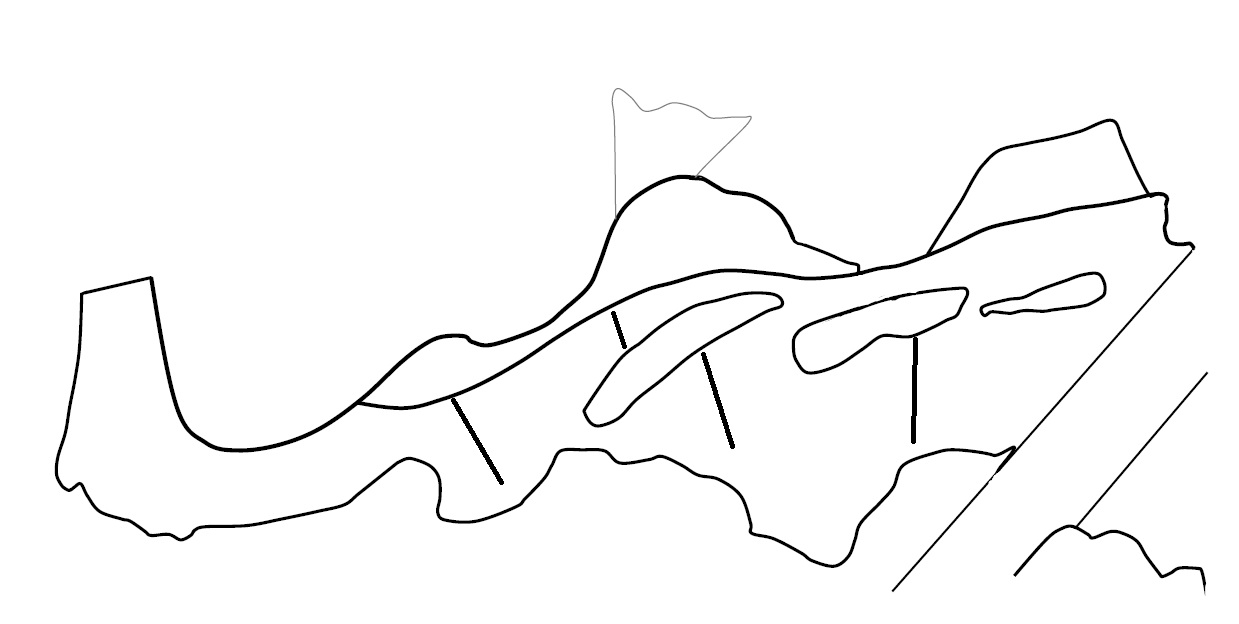

Supplement: S1 Fig — This jpg file contains the hand-drawn map used in data collection and processing. (JPG) [file pone.0297687.s001.jpg]

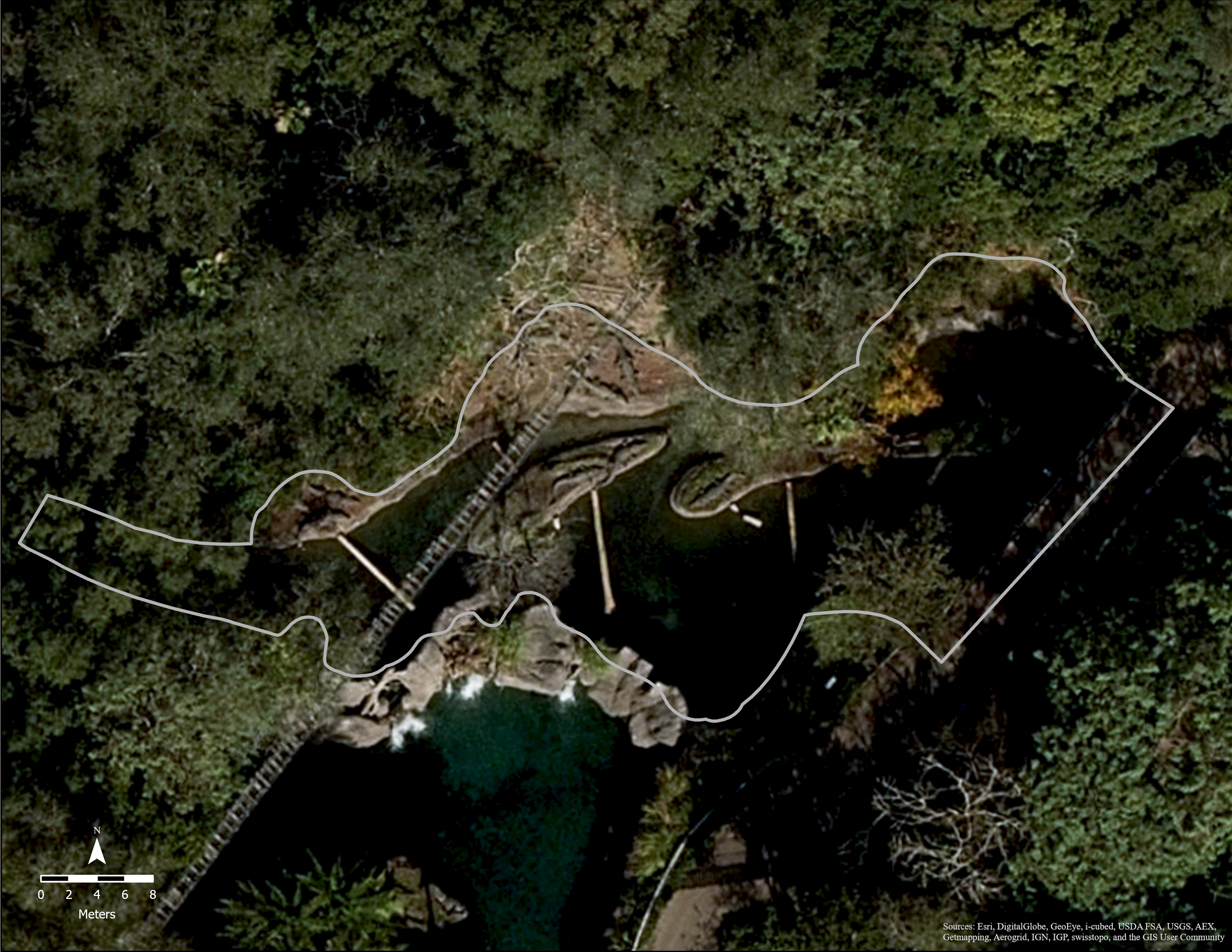

Supplement: S2 Fig — This jpg file contains an outline of the crocodile exhibit over satellite imagery. This map was created using ArcGIS® software by Esri. ArcGIS® and ArcMap™ are the intellectual property of Esri and are used herein under license. Copyright © Esri. All rights reserved. For more information about Esri® software, please visit www.esri.com. (JPG) [file pone.0297687.s002.jpg]
