# Supplementary material for: Home is where the home range is: Identifying territoriality and exhibit preferences in an ex-situ group of all-male Nile crocodiles (Crocodylus niloticus)
Source: PLoS One. 2024 Jan 25;19(1):e0297687. doi: 10.1371/journal.pone.0297687 (PMC10810454; doi:10.1371/journal.pone.0297687)
Supplement: S1 Table — Generalized linear mixed model outputs for predictors of home range size (A), core area size (B), utilization distribution overlap index (UDOI) (C), degree during all seasons (D), degree during the cold season (E), and degree during the warm season (F). CA-CA refers to core area to core area overlap, HR-CA refers to home range to core area overlap, and HR-HR refers to home range to home range overlap. Bolded variables denotes statistical significance (P ≤ 0.05) for predictor variables. Parameter estimates with ‶-″ are compared to remaining variable conditions within each predictor variable. (DOCX) [file pone.0297687.s003.docx]

**S1 Table.** Generalized linear mixed model outputs for predictors of home range size (A), core area size (B), utilization distribution overlap index (UDOI) (C), degree during all seasons (D), degree during the cold season (E), and degree during the warm season (F). CA-CA refers to core area to core area overlap, HR-CA refers to home range to core area overlap, and HR-HR refers to home range to home range overlap. Bolded variables denotes statistical significance (P ≤ 0.05) for predictor variables. Parameter estimates with ‶-″ are compared to remaining variable conditions within each predictor variable.

| **Predictor Variable** | **X^2^** | **D.F.** | **P** | **Variable Condition** | **Estimate** | **S.E.** | **z** | **P** |
| --- | --- | --- | --- | --- | --- | --- | --- | --- |
| *(A) Home range size* | |  |  |  |  |  |  |  |
| Intercept | 0.031 | 1 | 0.860 | - | -11.974 | 67.911 | -0.176 | 0.860 |
| Season | **7.748** | **1** | **0.005** | Cold | - | - | - | - |
|  |  |  |  | **Warm** | **10.555** | **3.792** | **2.783** | **0.005** |
| Weight | 2.107 | 1 | 0.147 | - | 0.296 | 0.204 | 1.452 | 0.147 |
| *(B) Core area size* |  |  |  |  |  |  |  |  |
| Intercept | 0.001 | 1 | 0.975 | - | -0.394 | 12.693 | -0.031 | 0.975 |
| Season | 0.125 | 1 | 0.724 | Cold | - | - | - | - |
|  |  |  |  | Warm | -0.239 | 0.677 | -0.354 | 0.724 |
| Weight | 1.678 | 1 | 0.195 | - | 0.050 | 0.038 | 1.296 | 0.195 |
| *(C) UDOI* |  |  |  |  |  |  |  |  |
| Intercept | 18.052 | 1 | <0.001 | - | 0.061 | 0.014 | 4.249 | <0.001 |
| Season | **3.932** | **1** | **0.047** | Cold | - | - | - | - |
|  |  |  |  | **Warm** | **0.010** | **0.005** | **1.983** | **0.047** |
| Level | **1006.401** | **2** | **<0.001** | CA-CA | - | - | - | - |
|  |  |  |  | **HR-CA** | **0.029** | **0.007** | **4.341** | **<0.001** |
|  |  |  |  | **CA-CA** | **0.202** | **0.007** | **29.113** | **<0.001** |
| Difference in Weight 3.223 | | 1 | 0.073 |  | 0.0003 | 0.0002 | -1.795 | 0.073 |
| *(D) Degree* |  |  |  |  |  |  |  |  |
| Intercept | 810.050 | 1 | <0.001 | - | 2.309 | 0.081 | 28.461 | <0.001 |
| Season | **5.593** | **1** | **0.018** | Cold | - | - | - | - |
|  |  |  |  | **Warm** | **-0.100** | **0.042** | **-2.365** | **0.018** |
| *(E) Degree – cold season* | |  |  |  |  |  |  |  |
| Intercept | 23.859 | 1 | <0.001 | - | 3.078 | 0.630 | 4.885 | <0.001 |
| Level | **88.342** | **2** | **<0.001** | CA-CA | - | - | - | - |
|  |  |  |  | **HR-CA** | **0.439** | **0.078** | **5.648** | **<0.001** |
|  |  |  |  | **HR-HR** | **0.697** | **0.074** | **9.389** | **<0.001** |
| Weight | **3.881** | **1** | **0.049** | - | **-0.004** | **0.002** | **-1.970** | **0.049** |
| *(F) Degree – warm season* | |  |  |  |  |  |  |  |
| Intercept | 23.932 | 1 | <0.001 | - | 2.916 | 0.596 | 4.892 | <0.001 |
| Level | **74.705** | **2** | **<0.001** | CA-CA | - | - | - | - |
|  |  |  |  | HR-CA | **0.375** | **0.084** | **4.487** | **<0.001** |
|  |  |  |  | HR-HR | **0.676** | **0.079** | **8.568** | **<0.001** |
| Weight | 3.418 | 1 | 0.065 | - | -0.003 | 0.002 | -1.849 | 0.065 |
